# Comment on “Epidemiological Survey on Porcine Cysticercosis in Nay Pyi Taw Area, Myanmar”

**DOI:** 10.1155/2016/1624541

**Published:** 2016-01-12

**Authors:** Maria Teresa Galán-Puchades, Màrius Vicent Fuentes

**Affiliations:** Department of Cellular Biology and Parasitology, Faculty of Pharmacy, University of València, Avenida Vicent Andrés Estellés s/n, Burjassot, 46100 Valencia, Spain

We have read with interest the paper by Khaing et al. [1], in which first data on* Taenia solium* cysticercosis in pigs from Myanmar are published. The authors found a porcine cysticercosis prevalence of 23.67% in slaughtered pigs, which, as they mention, indicates the presence of human taeniasis and also the risk of acquiring human cysticercosis and, therefore, neurocysticercosis.

The high porcine cysticercosis prevalence detected by the authors means, obviously, that there has to be a high prevalence of human* T. solium* taeniasis among the inhabitants of Myanmar leading to a high presence of infective eggs in the environment. However, as far as we know, the presence of* T. solium* taeniasis, as well as neurocysticercosis, has only been diagnosed in refugees or immigrants from that country but not in people living in Myanmar [2–6].

There is no doubt that* T. solium* is present in Myanmar; however, our query is whether other human* Taenia* species might also be present in that country and, in particular, whether* T. asiatica* could be present in Myanmar as suggested more than 20 years ago by Fan et al. [7] and recently by Anantaphruti [2].

*T. asiatica* was described as a new species in 1993 [8]. This third human* Taenia* species has practically the same gravid proglottid morphology as* Taenia saginata*, but its life cycle is just the same as that of* T. solium*; that is, its intermediate hosts are pigs instead of cattle, the intermediate hosts for* T. saginata*. The clear liver tropism the cysticerci of* T. asiatica* presents in pigs is worth mentioning. Regarding its geographical distribution, the species has been found in Taiwan, South Korea, Philippines, Indonesia, Thailand, China, Vietnam, Japan, and Nepal [9]. Although* T. asiatica* was initially considered as an exclusively Southeastern Asian parasite, its finding in Nepal (far from Southeast Asia) and the phenomenon of globalization (it is a parasite with cosmopolitan hosts) have recently led to the notion that the species probably has a wider distribution [10, 11]. The presence of* T. asiatica* in three more Asian countries, Myanmar, Laos, and Malaysia, is highly suspected [9].

Concerning Myanmar, no cases have been confirmed so far, but a case identified as* T. asiatica* was detected in a Karen immigrant who moved from Myanmar to Kanchanaburi province, Thailand, in 2005. However, she had already started to expel proglottids in faeces in 1997, when she was still living in Myanmar [2]. Therefore, the presence of* T. asiatica* in Myanmar is very likely.

*T. asiatica* may be found in the definitive (humans) as well as in the intermediatehost (pigs). The detection of* T. asiatica* taeniasis is by no means an easy task. Taking into account that neither the characteristics of the eggs nor the morphology of the gravid proglottids are specific enough to distinguish* T. asiatica* from* T. saginata*, these two species can only be differentiated by means of molecular techniques (e.g., multiplex PCR (polymerase chain reaction)) [12]. Unfortunately, these expensive molecular methods are not normally employed in routine diagnosis.

A specific meat inspection should be carried out to detect* T. asiatica* cysticerci in pigs. Routine meat inspections conducted in pigs in Myanmar include the study of* T. solium* predilection sites such as the tongue, masseter, brain, shoulder, diaphragm, heart, and skeletal, fore limb as well as hind limb muscles [1]. Considering that the small cysticerci of* T. asiatica* are almost exclusively located in the liver, such local inspections would not be sensitive enough to detect infected livers [13]. In addition,* T. asiatica* cross-reacts even in the most specific immunological method to detect* T. solium* cysticercosis, the enzyme-linked immunoelectrotransfer blot (EITB) [14]. Therefore, serological tests would currently not be specific enough to detect* T. asiatica* cysticercosis.

For the aforementioned reasons, we urge Khaing and colleagues to take advantage of their research in pigs in Myanmar and carry out a detailed study of the surface as well as the parenchyma of pig livers.* T. asiatica* cysticerci can easily be differentiated from those of* T. solium* due to their smaller size as well as the lack of hooklets or the presence of vestigial ones [8]. Molecular techniques, however, would be the key to ascertain whether* T. asiatica* is definitely present in Myanmar or not.
